# COVID-19 and Psychosocial Changes: Results From the National Health and Aging Trends Study (NHATS)

**DOI:** 10.1093/geroni/igab046.2159

**Published:** 2021-12-17

**Authors:** Laura Samuel, Anthony Ong

## Abstract

The COVID-19 pandemic likely altered many aspects of daily life for older adults, including social connectedness, technology use, financial resources and hopefulness. This symposium examines these exposures and changes during the COVID-19 pandemic and tests their associations with health and related factors. Analyses are all conducted among a nationally representative sample of U.S. adults aged ≥65 years who participated in the NHATS COVID-19 supplement, which was a mail-in survey with participant and proxy respondents conducted between June and October of 2020. Additional NHATS participant data collected between 2011 and 2019 was used to account for individual characteristics before COVID-19, including demographic, socioeconomic and relevant health characteristics. Sampling weights were applied to analyses to account for study design and non-response so that inferences can be drawn to the US population of adults aged ≥65 years. This symposium will present results from five COVID-19 pandemic focused studies that examine the associations between 1) financial changes and health, 2) loneliness and behavioral changes, 3) hopefulness with function, sleep and loneliness, 4) technology use and mental health, and 5) predictors of technology use. These results offer insights into the mechanisms that influence health during the COVID-19 pandemic. Results have clinical, policy and public health implications because they can inform the development of interventions, programs and policies with potential to improve health and health care and advance health equity for older adults.

